# Electromyographic Comparison of an Abdominal Rise on a Ball with a Traditional Crunch

**DOI:** 10.3390/s22051979

**Published:** 2022-03-03

**Authors:** Aleš Dolenec, Mojca Svetina, Vojko Strojnik

**Affiliations:** Faculty of Sport, University of Ljubljana, 1000 Ljubljana, Slovenia; mojca.svetina.curly@gmail.com (M.S.); vojko.strojnik@fsp.uni-lj.si (V.S.)

**Keywords:** strength exercise, muscle recruitment, EMG, abdominal muscles

## Abstract

We propose a new exercise, the abdominal rise on the ball, to replace the traditional crunch in exercise programs. The aim of this study is to compare the activity of the abdominal muscles when performing an ARB with the same activity when performing a traditional crunch. Twenty healthy adults participated in the study. Surface electromyography (EMG) was recorded from the upper and lower rectus abdominis (URA, LRA), internal oblique (IO), external oblique (EO), transversus abdominis (TrA), and erector spinae (ES). EMG values were normalized to maximal voluntary isometric contraction. A paired *t*-test, nonparametric Wilcoxon test and correlation coefficient were used for statistical analysis. The normalized EMG values of EO, TrA and ES, were statistically significantly higher during the abdominal rise on the ball compared to the traditional crunch, while URA, LRA and IO were significantly lower during the abdominal rise on the ball compared to the traditional crunch. TrA, EO and IO are sufficiently activated during an abdominal rise on a ball, so the exercise could be deemed effective for strengthening these muscles.

## 1. Introduction

The strengthening of the abdominal muscles is used in many exercise programs to improve core stability, reduce lower back pain, improve posture, and enhance athletic performance. In the literature, the most commonly used exercise is a TC, in which the legs are bent 90 degrees at the knees and 45 degrees at the hips. During the exercise, the torso bends up to 30 degrees. The differences between the exercise variations are mainly in the position of the arms, which can be behind the head, crossed on the chest, stretched along the torso or placed on the thighs. In the TC exercise, the arm position acts as a load. Electromyography (EMG) studies have shown that a TC primarily activates the rectus abdominis muscle, with the external oblique muscle (EO) and internal oblique muscles (IO) acting as synergists [1,2,3,4,5], with little activation of the transversus abdominis muscle (TrA). During a TC, the spine is flexed and extended, causing relatively high local pressure on the vertebrae and intervertebral discs [2]. In addition, the walls of the intervertebral discs can be damaged by repeated flexion [6]. The latter is especially true if the damaged walls of the discs are not given sufficient time to regenerate [7]. Due to the activation and consequent strengthening of the RA muscle in a TC exercise, this exercise is important if we want to improve the stabilization of the core in the sagittal plane. Core stability in the sagittal plane is only one part of the core stability needed in our daily activities. Therefore, the TC cannot satisfactorily improve core stability in multiple planes [8,9].

Spinal stability can be improved by intra-abdominal pressure (IAP) [10,11,12]. IAP can also reduce spinal loading [13]. Activation of the transversus abdominis muscle has been associated with IAP [14]. However, the study on the activation of the abdominal muscle during the common modifications of TC showed that TC may not be suitable as a strength-training exercise for this muscle due to the low activation level of the TrA [15]. In addition, IAP decreases during a TC as it counteracts trunk flexion [16].

Many studies have compared abdominal muscle activity during different exercises with or without equipment. In most, a TC was used as the basis of comparison because it is the most widely used and universal exercise for strengthening the abdominal muscles [1,2,3,4,5,17,18,19]. In many training programs a TC was the only exercise that involved the abdominal muscles. As a supplement to a TC or as a replacement for a TC in programs with a single exercise for the abdominal muscles, in order to gain more effective stabilization of the core and reduced spinal loading, we propose an exercise that could activate the abdominal muscles associated with the IAP without excessive loading of the spine and disks. We called this exercise the abdominal rise on the ball (ARB).

ARB reflects an attempt to provide concentric and eccentric contractions for the abdominal muscles while maintaining the spine in a neutral position. The body is kept in a prone position during the exercise. A ball at the level of the navel supports the body. By activating and relaxing the abdominal muscles to control IAP, the body is raised and lowered on the ball. IAP is a hydraulic pressure that works in all directions. When you lie prone on the ball, your body weight pushes your abdominal wall against your spine. As you increase IAP, the outward tension shifts the abdominal wall outward [20] and lifts the body. This means that the abdominal muscles work as in dynamic exercises, while the spine is loaded as in static exercises and maintains its neutral posture. The IAP can relieve the spine longitudinally to a considerable extent [13], so we assume that, despite the activity of the trunk extensor muscles, the pressure on the intervertebral discs is tolerable. The neutral posture of the spine distributes the load evenly among the vertebrae and intervertebral discs, reducing the risk of high local pressure [2] and injury [6]. The latter may be of particular interest in osteoporosis patients to prevent anterior collapse of the vertebrae [21].

The aim of the present study was to test the hypothesis that an ARB activates the muscles associated with the IAP more than a TC and that the muscles associated with the IAP are the prime movers in ARB. For this purpose, we compared the EMG activity of the upper rectus abdominis muscle (URA), lower rectus abdominis muscle (LRA), internal oblique muscle, external oblique muscle, transversus abdominis, and erector spinae muscle (ES) during a traditional crunch and an abdominal rise on a ball.

## 2. Materials and Methods

### 2.1. Design and Participants

Twenty healthy physical education students (10 males and 10 females) volunteered to participate in this study. The mean (±SD) for age, height, and body mass was 22 (±3.9) years, 181.3 (±7.6) cm, and 75.9 (±9) kg for males and 22 (±2.7) years, 167.6 (±4.5) cm, and 57.5 (±4) kg for females. All subjects signed a faculty-approved informed consent form. All subjects were physical education students. Subjects were visually selected to ensure that they had sufficient subcutaneous adipose tissue to allow for the accurate measurement of muscle activity. For inclusion in the study, the volunteers had to be able to do at least 8 reps of the ARB and TC, and had to have no history of low back pain. Subjects were excluded from the study if they presented contraindications to the exercises proposed in this study, had a chronic disease or were using drugs of any description during the measurements. Women had to be without menstruation during the measurements. Because abdominal muscle activity patterns are similar in male and female subjects [5], we did not separate the data by sex.

Each subject completed six training sessions over three weeks. During the sessions, subjects practiced the correct execution of the exercises and timing of repetitions. On the test day, a standardized warm-up protocol was performed; then, the measurement equipment was placed on the subjects. Before maximum voluntary isometric contraction (MVIC) was measured, subjects performed two activation exercises. MVIC was measured twice for each muscle. For data-collection purposes, subjects performed 1 set of 5 repetitions for each abdominal exercise. The testing order of ARB and TC was randomized for all subjects, and all data for each subject and exercise were collected in a single day.

To ensure temporal consistency, each subject was instructed to perform each movement at a constant speed during the concentric and eccentric phases. A metronome was used to time each phase of the movement at a rate of 1 and 2 s per phase (concentric and eccentric, respectively). Sufficient rest of more than 3 min was provided between trials to avoid fatigue. None of the subjects showed signs of fatigue at any time during data collection.

### 2.2. Instruments

Six muscles were used for data acquisition: URA, LRA, IO, EO, TrA, and ES. Disposable surface electrodes (Ag/AgCl Kendall, Neustadt/Donau, Germany) were placed on the right side of the body, except for TrA where these were placed on the left side of the body, as EMG electrodes were placed similarly to IO. Placement of electrodes for URA was measured at 25% of the proximal distance between the xiphoid process and the pubic symphysis which is midway between the lateral border of the rectus abdominis and the linea alba [22]. On the LRA, the electrodes were placed 8 degrees from vertical in an inferomedial direction and centered on the muscle belly near the midpoint between the umbilicus and the pubic symphysis and 3 cm laterally from the midline [23]. The electrodes were placed over the EO and measured two finger widths above the anterior half of the iliac crest [22]. Electrode placement for the IO was horizontally 2 cm inferomedial to the ASIS, within a triangle bounded by the inguinal ligament, the lateral edge of the rectus sheath, and a line connecting the two ASIS, with only the aponeurosis of the external oblique muscle (and not the external oblique muscle) covering the IO [24,25]. For the TrA, electrodes were placed 2 cm cephalic of the pubis and lateral to the midline, and parallel to the ramus pubicus superior [26]. The electrodes for the erector spinae were placed 1 finger width medial to the line from the posterior superior iliac spine, to the lowest point of the inferior rib at the level of L2 [27]. The ground electrode was placed on the patella [27]. The electrodes were aligned parallel to the muscle fibers with a distance of approximately 2 cm between the electrodes. Before placing the electrodes, the skin under each electrode was shaved and cleaned with alcohol to reduce the impedance at the interface between the skin and the electrodes. EMG signals were sampled at 2000 Hz per channel. The PowerLab system (AD Instruments, Dunedin, New Zealand) was used to transfer the signals to a laptop computer for analysis (LabChart 7 software, AD Instruments, Dunedin, New Zealand, 2014).

### 2.3. Exercises

The standardized warm-up protocol consisted of an eight-minute run and two activation exercises (eccentric-concentric contraction). The first exercise was a very rapid torso twist left and right with a small amplitude (4 consecutive repetitions on each side). The second exercise was a very rapid swing of the arms down and up in front of the body (5 separate repetitions with a short break between repetitions). In both exercises, the subject held a medicine ball weighting 2 kg in their hands.

**Figure 1:**
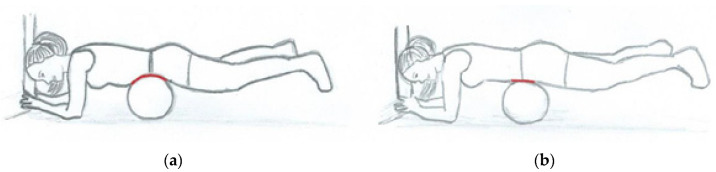
Abdominal rise on the ball: (**a**) starting position: the ball intrudes into the abdominal region (see the red curve); (**b**) ending position: the ball is pushed out of the abdominal region (see the red line).

The starting position at ARB was prone. At the level of the navel, a basketball supported the body with the rest of the body in the air. The ball was fixed to the floor so that it did not move during the exercise. The participant held onto a stable object with both hands so that the weight of their legs did not overload the rest of the body and force their feet to touch the ground (Figure 1a). The abdominal muscles were relaxed so that the ball intruded into the abdominal region (see the red curve in Figure 1a). By activating the abdominal muscles, the participant pushed the ball out of the abdominal cavity so that the body rose on the ball (see the red line in Figure 1b) and reached its final position. The spine remained in a neutral position during the exercise. The target time was 1 s for raising (concentric contraction) and 2 s for lowering the body (eccentric contraction) so that there was a smooth movement, without an abrupt start.

The TC was performed in accordance with the American College of Sports Medicine guidelines. Subjects began in the supine position with their knees bent 90 degrees, placing the tips of the middle fingers of both hands on a marked starting line. Subjects then raised their torsos and slid their hands horizontally along the mat until the tips of their middle fingers reached the 10-cm line. Subjects immediately returned to the starting position in a slow, controlled manner. Again, the time target was 1 s for raising (concentric contraction) and 2 s for lowering (eccentric contraction).

### 2.4. Data Analyses

To normalize the EMG signals, maximal voluntary isometric contractions of the selected abdominal muscles were performed by the participants before the exercises. During the exercises, only concentric contractions were analyzed. When processing the data from MVIC and the exercises, the signal was digitally filtered (20 Hz/500 Hz, bidirectional) to remove the baseline shift. Later, the signals were full wave rectified. Within the 1 s concentric contraction of the abdominal muscles, the middle 500 ms were averaged and used for further analysis. We then averaged the 2nd, 3rd, and 4th repetition and normalized these to MVIC for each exercise.

### 2.5. Statistical Analyses

Statistical analyses of the normalized EMG values were performed using IBM SPSS version 25 (IBM, Armonk, NY, USA). Inter-class correlation (ICC) (Cronbach α) was calculated for each variable for three measurements (2nd, 3rd, and 4th repetition). For all variables, the Shapiro–Wilk test was used to check for normal distribution. For normally distributed variables (*p* > 0.05), a dependent *t*-test was performed on each muscle for two loading situations. For non-normally distributed variables, a Wilcoxon signed-rank test was used. Correlation coefficients were calculated for the IO and TrA muscles. Statistical significance was accepted at an alpha level of 0.05 (two-sided test). Effect size was defined according to Cohen [28] as small (<0.30), medium (0.30–0.49), and large (>0.50).

## 3. Results

**Figure 2:**
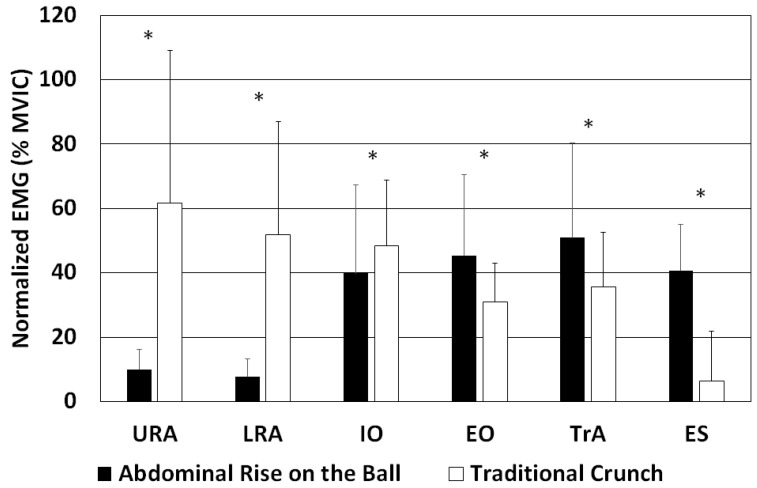
Normalized EMG values and SD for the upper rectus abdominis (URA), lower rectus abdominis (LRA), internal oblique (IO), external oblique (EO), transversus abdominis (TrA), and erector spinae (ES) muscles in an abdominal rise on a ball and a traditional crunch. * *p* ≤ 0.05.

The averaged normalized EMG values and SD are shown in Figure 2. All variables had a high ICC, ranging from 0.995 to 0.847. The IO variable was the only one distributed normally. A dependent *t*-test was used to compare this variable between the ARB and TC exercises. Other variables (URA, LRA, EO, ES, and TrA) were not distributed normally, so the Wilcoxon signed-rank test was used to compare the ARB and TC exercises. For an ARB, URA, LRA, IO, EO, TrA, and ES, the activity was 10 ± 6%, 8 ± 6%, 40 ± 27%, 45 ± 25%, 51 ± 29%, and 41 ± 14%, respectively, whereas for a TC, URA, LRA, IO, EO, TrA, and ES, the activity was 62 ± 47%, 52 ± 35%, 48 ± 20%, 31 ± 12%, 35 ± 17%, and 6 ± 15%, respectively. The activities of EO, TrA and ES were significantly higher, while the activities of URA, LRA and IO were significantly lower when undertaking an ARB compared with a TC. Because a TC is the standard to which ARB was compared, the EMG values for URA, LRA, IO, EO, TrA, and ES during a TC were assigned a value of 100%, and the activities of each muscle were expressed for ARB relative to TC (Table 1). The effect sizes of the differences in muscle activity between ARB and TC ranged from moderate to large (Table 1). The correlation coefficient between IO and TrA was 0.065 (*p* > 0.05) and 0.154 (*p* > 0.05) for ARB and TC, respectively.

## 4. Discussion

A comparison of a TC and an ARB showed that there were significant differences in muscle activity during the exercises. When performing a TC, the URA, LRA, and IO muscles were more active, whereas the EO, TrA, and ES muscles were more active when performing an ARB. Normalized muscle activity ranged from 10 to 62% MVIC. The most activated abdominal muscles when performing a TC were URA, LRA and IO muscles, whereas when performing an ARB they were the TrA, EO, and IO muscles. Choi, Kim and Cynn [17] represented the activity of IO and TrA as a common activity because they assumed that a certain placement of EMG electrodes would reflect the EMG activity of both muscles simultaneously. In our study, IO and TrA were measured at different positions [26]. We calculated the correlation coefficient between the EMG activity at both positions and found that the correlation was low and statistically insignificant. Therefore, we conclude that the measured EMG activity at these two positions reflects the EMG activity of different muscles. In both exercises, the abdominal muscles perform concentric and eccentric contractions. An important difference between the exercises is that in an ARB the spine always remains in a neutral position, while in a TC, the lumbar spine is flexed and extended.

In our study, URA and LRA were the most active muscles when performing a TC, which is consistent with the results of other studies [3,4,9,15,18,23,29] and appears logical because URA and LRA are the major trunk flexors. When performing an ARB, these two muscles were the least active. During static trunk extension, their low activity is necessary because the muscles are antagonists during static trunk extension [30]. Therefore, their increased activity in maintaining the static position of the trunk would require increased activity of the trunk extensors. Increased activity of the trunk extensors increases the compressive force on vertebrae and intervertebral disks [9,31,32,33], which is not always desirable. When performing an ARB, the rectus abdominis might contribute some trunk-lifting force at the onset of concentric contraction if the abdomen is concave due to the pressure of the base of the ball and part of the muscle’s force vector is in the direction of the trunk lift. If you align the direction of action of the muscle (straight line between the muscle attachments), then this part of the force vector disappears. As the abdomen extends beyond this line, the force vector of the rectus abdominis muscle begins to lower the trunk, which no longer corresponds to the task of lifting the body. In addition, activation of the rectus abdominis increases the compressive forces in the spine [9].

When performing an ARB, the lifting of the body is due to the coordinated work of the IO, EO, and TrA muscles, which form the abdominal wall at the front of the trunk and, together with the pelvic floor muscles and diaphragm, control intra-abdominal pressure (IAP) [10,20,34]. Due to the high activity levels of IO, EO, and TrA in the ARB, it can be argued that the IAP is large during exercise and can reduce or even neutralize the influence of EC on the compressive force in the lumbar spine in the upright body position, as shown by Stokes, Gardener-Morse, and Henry [35]. When performing an ARB, the body weight pushes the ball toward the spine, increasing the IAP and causing the relaxed abdominal muscles to move toward the spine and probably slightly to the side. This results in a stretching of the abdominal muscles. Because of the IAP, the diaphragm is likely to rise slightly. Activation of the abdominal wall muscles, likely via increased IAP [10,35,36,37,38], and formation of the abdominal muscles into a solid support [37,39], raises the body. In this process, the anterior muscles of the abdominal wall perform a concentric contraction. Their potential to lift the trunk is related to the pressure in the abdominal cavity, as shown by the fact that they can be lifted above the line defined by the pubis and sternum without involving the rectus abdominis muscle, whose activation is typically associated with exercises in which it is necessary to reduce the pressure in the abdominal cavity [40], as is also shown in this study when performing a TC.

The ES must be active during an ARB as the ES ensures that the legs are not supported and are lifted off the ground during exercise through isometric contraction control [41]. The measured EMG values showed that the ES was active when performing an ARB. In contrast, the ES muscle was mostly inactive when performing a TC, which is consistent with other studies [18,42]. This was expected because the ES muscle acts as an antagonist to the URA and the LRA muscles when performing a TC.

From the point of view of loading the spine or increasing the compressive force, an ARB could initially be considered unsuitable, since the ES is more active when performing an ARB than when performing a TC. In fact, the ES muscle essentially increases the compressive force in the lumbar spine [31,32]. However, in combination with increased IAP, compressive forces in the lumbar spine may be reduced despite increased ES activity [32,35,43,44]. Since the ARB involves the IAP, it can be concluded that an important part of the compressive force on the spine caused by the activation of ES is reduced. At the same time, the spine is kept more or less static and in a neutral position. The neutral position of the spine allows for the transmission of compressive forces to the entire surface of the vertebrae or intervertebral discs, significantly reducing localized pressure, which is one of the main causes of spinal injury. This is especially important for people who already have problems with the intervertebral discs and for people with osteoporosis [21,45]. Although the activation of ES is much lower when performing a TC than an ARB, a TC may be a risky exercise for patients with osteoporosis because of the high local load on the intervertebral discs and vertebrae. In this case, the rectus abdominis assumes the role of ES in the sense that it exerts pressure on the spine while there is little or no IAP support and, at the same time, the area of force transmission is smaller due to the flexed spine, which increases the local load on the intervertebral discs and vertebrae. Therefore, torso-bending exercises are contraindicated in people with osteoporosis [45].

Depending on the level of muscle activation (31 to 51% MVIC), both a TC and an ARB have the potential to strengthen muscles even without additional load. The main effect of performing these exercises would be to improve strength endurance. An ARB seems to be better for strengthening the TrA muscles than a TC. In addition to the greater activity of the TrA muscles and other IAP-supporting muscles, an ARB has the advantage over a TC due to a more even loading of the spine and activation of the pelvic floor and diaphragm muscles due to increased IAP [10,44]. From a practical point of view, when a limited numberof exercises is considered, we believe that an ARB has an advantage over a TC because it is associated with muscular training that provides greater core stability and does not cause local load on the spine. If the trainee’s interest is in specific trunk flexor training, then the TC exercise is the better choice. Otherwise, both exercises can be performed simultaneously for more complex core stability control.

As with most studies analyzing calisthenic exercise, there were some limitations to our study, including the fact that we did not know the exact load exerted in each exercise and by each subject. This could lead to some bias in the results. The next limitation arose from the selection of the subjects who were healthy, young adults with no lower back pain. For this reason, the transfer of the results to other populations should be further tested. In the future, it would be useful to conduct a study of the effects of an ARB in different groups of people, such as those with lower back pain and those who are frail, and to more precisely define the exercise load.

## 5. Conclusions

An ARB is an exercise that might be considered as a substitute for a TC if the goal of the exercise is to strengthen TrA, IO, EO, that is, if core stability is a goal. An ARB is of particular interest to those who have limited motion in the lumbar spine, as there is no movement in the spine and the spine is in a neutral position at all times. Another unique feature of an ARB is that URA and LRA are not activated, which means that the load is placed directly on TrA, IO and EO, unlike most abdominal exercises where the primary antagonist is URA and LRA, while IO, EO and TrA are only synergists.

## Figures and Tables

**Table 1:** Percentage difference in normalized EMG in an abdominal rise on a ball relative to a traditional crunch (*N* = 20).

Muscle (%)	Abdominal Rise on the Ball	Traditional Crunch ^1^	Effect Size
Upper rectus abdominis (URA)	16	100	0.85
Lower rectus abdominis (LRA)	15	100	0.82
Internal oblique (IO)	82	100	0.67
External oblique (EO)	146	100	0.37
Transversus abdominis (TrA)	143	100	0.31
Erector spinae (ES)	641 ^2^	100	0.77

^1^ Because the traditional crunch is the standard to which the abdominal rise on the ball was compared, the EMG values for the URA, LRA, IO, EO, TrA, and ES during the traditional crunch were assigned a value of 100%. ^2^ The excessive value is due to the inactivity of this muscle in TC.

## Data Availability

All the data and reported results are available on request to the corresponding author.
